# Development of a RP-HPLC Method for Analysis of *Triphala Curna* and its Applicability to Test Variations in *Triphala Curna* Preparations

**DOI:** 10.4103/0250-474X.57286

**Published:** 2009

**Authors:** V. Pawar, P. Lahorkar, D. B. Anantha Narayana

**Affiliations:** Unilever Research India, 64 Main Road, Whitefield, Bangalore-560 066, India

**Keywords:** *Triphala Curna* (TC), gallic acid, chebulagic acid, chebulinic acid, myrobalans

## Abstract

A sensitive, rapid, reverse phase HPLC method is reported for analysis of *Triphala Curna* using gallic acid, chebulagic acid and chebulinic acid as markers. Validation data for the method has been provided. Unlike methods of recovery testing adopted for synthetic chemicals, a modified approach has been presented here for a formulation like *Triphala Curna* having three myrobalans in its composition. Data has been provided to demonstrate applicability of the method developed to assess the variation in the *Triphala Curna* preparations.

*Triphala Curna* is one of the well known powdered preparations in Indian system of health care, Ayurveda since ancient time[1]. TC finds extensive usage both by way of prescription by Ayurvedic doctors and as an OTC preparation. The product is available in both ayurvedic pharmacies and also in health food stores. The product comprises of a coarse powder made out of three myrobalans, *Emblica officinalis* Gaertn (*Amla*), *Terminalia chebula* Retz. (*Haritaki*) and *Terminalia belerica* Roxb. (*Bibhitaki*) blended in equal proportion. From now onwards in this communication, these three myrobalans have been referred to as a*mla, belerica* and *chebula*. Currently compresses tablets of Triphala preparation are also available for the consumers. These tablets are prepared by mixing aqueous extracts of Triphala herbs to reduce the variability of dose which may not be possible to achieve in powder preparation. More than one hundred firms in India produce this preparation. TC is used to promote immunity[2]. It improves digestion and assimilation, corrects constipation, cleanses and tonifies the gastrointestinal tract[3]. TC and its constituents act as cardio-tonic, control blood pressure, improves blood circulation and reduces cholesterol levels[4]. TC shows immunomodulatory properties and helps in improving the body's defense system[5]. In recent years there are several reports in the literature which suggest that TC possesses antimutagenic, radioprotecting and antioxidant activity[6–8].

Most Ayurvedic formulations are polyherbal. For such polyherbal preparations, it is necessary to validate the uniformity of mixing or blending in appropriate proportion. Here the challenge lies in estimating individual herbal components quantitatively and can provide guidance to identify variations in mixing or blending. Such studies have not been so far reported. We have attempted to generate data and propose an approach for ayurvedic preparation (TC). TC as per process given in Ayurvedic Formulary of India (AFI) is prepared by mixing a 1:1:1 mix of ground dry fruits of *Amla, Belerica* and *Chebula*, also known as myrobalans. Very few methods have been reported for analyzing TC[9–12] such as UV spectroscopic method and HPTLC methods using gallic acid as marker. Recently a validated HPLC method has been published which quantifies five different markers in TC^[13]^, but does not deal with analysis of the composition of each of the herbs. There is considerable variation in levels of chosen marker compounds in individual herbs of TC preparation. Single marker will be specific to single herb and may not be application to test the variation in the TC preparation. To address such issue, we have developed a new HPLC method for analysis with three marker compounds as gallic acid, chebulagic acid and chebulinic acid. RP-HPLC method has been validated and further evaluated for its applicability to test variation in TC preparations. This paper deals with TC and is not applicable for tablets or capsules made out of TC or its extracts.

## MATERIALS AND METHODS

Gallic acid was obtained from Sigma-Aldrich (USA). Chebulagic acid and chebulinic acid were procured from M/s Natural Remedies, Bangalore. Peak purity of these markers was checked before analysis (peak purity minimum 90%), HPLC-grade acetonitrile was obtained from Merck. Potassium dihydrogen phosphate (GR grade) and orthphosphoric acid (LR grade) were obtained from Merck. Reverse phase C_18_ HPLC column was procured from Phenomenex. Ultra pure water was obtained by means of a Millipore (MilliQ apparatus)

### Herbs collection and sample preparation:

Individual herbs were purchased from four different geographies namely (Bangalore, Delhi, Mumbai and Hyderabad). The herbs were authenticated by a qualified botanist in house and specimens were preserved. Each herb was powdered using stainless steel domestic grinder to get powders (> 95% passed through 60#). These powders were used for extraction. Initially *amla*, *belerica* and *chebula* powders were weighed separately and extracted. TC was prepared by mixing *amla, belerica* and *chebula* powders in 1:1:1 proportion. To test our approach, three more non-ayurvedic herbal mixtures were prepared in a proportion as described in Table 1. Clean and dry glass pestle and mortar were used for mixing.

**TABLE 1:** RATIO OF *AMLA, BELERICA* AND *CHEBULA* TAKEN TO MAKE THE DIFFERENT HERBAL MIXTURES

Mix Code*	Ratio		
	
	*Amla* (a)	*Belerica* (b)	*Chebula* (c)
abc-111	1	1	1
abc-021	0	2	1
abc-102	1	0	2
abc-210	2	1	0

The table gives ratios of *amla, belerica* and *chebula* mixed to give different non-Ayurvedic formulations (abc-021, abc-102 and abc-210) and TC preparation as abc-111 where ‘a’ stand for *amla*, ‘b’ stand for *belerica* and ‘c’ stand for *chebula*. These formulations were analysed by HPLC to evaluation test variations in TC preparations.

### Extraction procedure:

The solvent mix for extraction was prepared by dissolving 0.136 g of potassium dihydrogen phosphate in 800 ml of water. 0.5 ml of orthphosphoric acid was added and shaken for 5 min to get homogenous solution. Volume was made up to 1l using acetonitrile. To prepare the TC and other blends with various proportions, each individual herb was accurately weighed. Each blend (50 mg) was taken in separate conical flasks. Extraction solvent (30 ml) was added to weighed blends and sonicated for 20 min at 27±3° in ultra sonicator water bath. The solution was centrifuged at 10,000 rpm for 15 min. Supernatant was collected and used for HPLC injection. Each extraction was done in duplicate and each extract was put for HPLC analysis in duplicate.

### Chromatographic conditions:

Chromatographic separation was performed on Shimadzu liquid chromatographic system equipped with LC 10AT *vp* solvent delivery system, SPDM-10Avp Photodiode array detector, SIL 10Avp auto-injector with cooler and 10Avp column oven. Class vp 6.01 Data station were applied for data collection and data processing. Phenomenex Luna C_18_(2) column (250×4.6 mm id) 5 micron was used for separation. Mobile phase A was 0.136 g of potassium dihydrogen phosphate dissolved in 1l of water. orthphosphoric acid (0.5 ml) was added shaken to get homogenous solution. Mobile Phase B was HPLC grade acetonitrile. Detection was done at 270 nm. Mobile phase was filtered through 0.45 micron membrane filter and degassed. Analysis was performed at 40°. Injection volume was kept at 20 μl with total flow rate of 1.5 ml/min for elution. System suitability was confirmed by performing all analysis. HPLC analysis was carried out by using a gradient elution in 0-18 min with 5-45% B, 18-25 min with 45-80% B, 25-28 min with 80-80% B, 28-35 min with 80-45% B, 35- 40 min with 45-5% B and 40-45 min with 5% B.

### Standard preparation and calibration:

Calibration curve was generated to quantify Gallic acid, chebulagic and chebulinic acid in samples. Five dilutions of each standard, at concentrations ranging from 2 to 40 μg/ml were prepared to generate calibration curve. Each standard was run in triplicate. The corresponding peak areas were plotted against the concentration of each of the marker under study.

### Accuracy and precision:

The accuracy of the method was determined by recovery experiments. TC samples were spiked with three different amounts of standard compounds prior to extraction. The spiked samples were extracted three times and analyzed as per above described method. From the data, percentage recovery and the relative standard deviation of recovery was calculated.

The precision of method was demonstrated by intra day and inter-day variation studies. In the intra day studies six replicate injections of standards and samples solutions were made and the response factor of standard compounds and percentage RSD were calculated. In the inter day variation studies six replicate injection of standard samples solutions were made and the response factor of standard compounds and percentage RSD were calculated.

### Linearity and range:

Linearity of the method was determined at five concentration levels of each standard ranging from 2 to 40 μg/ml. Each standard was run in triplicate the linearity of the detector response for the prepared standards was assessed by means of linear regression with respect to the amounts of each standard, measured in μg, and the area of the corresponding peak from the chromatogram.

### Limits of detection and quantification:

Limit of detection (LOD) and limit of quantification (LOQ) of the developed method were determined by injecting progressively low concentrations of the standard solutions using the developed RP-HPLC method (signal to noise ratio of 3) the LOD of gallic acid, chebulagic and chebulinic acid was found

### Robustness:

The robustness of the method was determined by making slight changes in the chromatographic conditions (flow rates, column dimensions and gradient variation). It was observed that there was no marked change in the chromatograms. The ruggedness of the method was determined by repeating the experiments on Agilent HPLC system by different operators in addition to Shimadzu.

### Selectivity and peak purity:

For the selectivity test a diode array detector was applied. The test was carried out on standard compounds, individual herb and TC extracts. The test was carried out using peak purity judgment, which is part of the Shimadzu Class LC10 software for handling 3D chromatographic data. The principle of peak purity judgment is to obtain three spectra, one at 50% up slope, one at the apex, and one at 66% down slope of individual peaks. These three spectra are compared, aiming for a similarity index as close to 1.0000 as possible. The peak purity was studied in the major peaks.

## RESULTS AND DISCUSSION

**Fig. 1:**
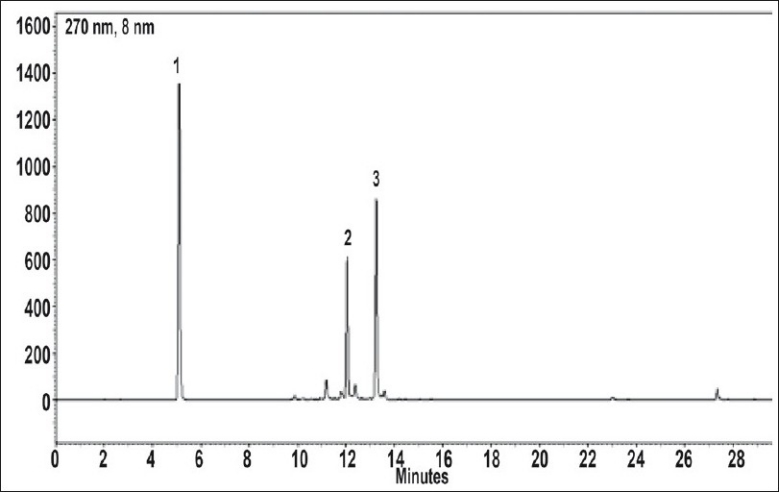
Representative HPLC chromatogram of standard marker compounds. Chromatogram shows gallic acid (1) peak at Rt- 5.20 min, chebulagic acid (2) peak at Rt - 12.15 min and chebulinic acid (3) peak at Rt - 13.30 min.

Under the chromatographic conditions employed, standard compounds have shown sharp peaks and good resolution (fig. 1). The HPLC method was validated by defining the linearity, peak purity, LOD, LOQ, precision, accuracy, specificity and robustness (Table 2). This validated method was used to analyse the samples.

**TABLE 2:** METHOD VALIDATION DATA

Compound	RT (min)	r^2^	Range (μg/ml)	LOD (μg/ml)	LOQ (μg/ml)	% Recovery (n=5)
gallic acid	5.20±0.12	0.999	20-400	0.49	1.98	102.33±1.25
chebulagic acid	12.15±0.17	0.998	20-400	0.48	1.94	102.18±0.99
chebulinic acid	13.30±0.11	0.989	20-400	0.52	2.07	101.45±0.65

The table gives mean retention times for each marker, regression coefficient (r^2^) from the calibration curve, limit of detection (LOD), limit of quantification (LOQ) and mean percent recovery, calculated for each marker (n=5).

**Fig. 2:**
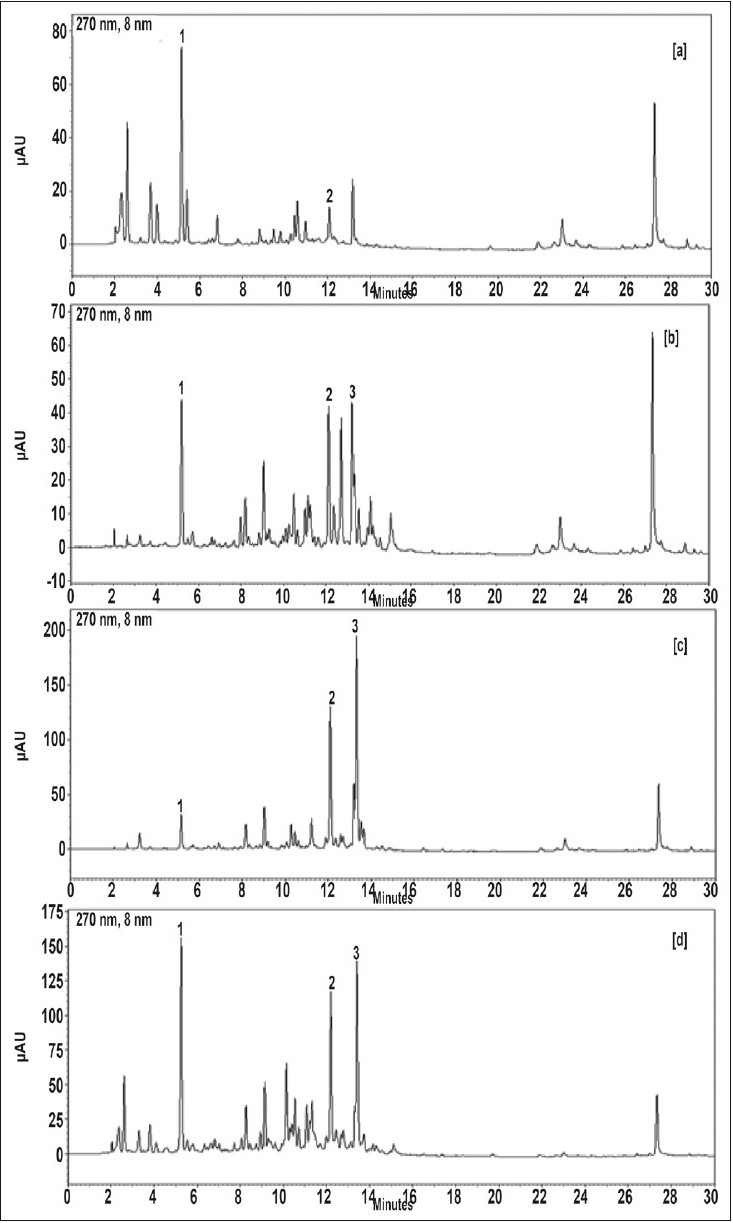
Chromatogram for *amla* [a], *belerica* [b], *chebula* [c] and *triphala curna* [d]. Chromatogram shows gallic acid (1) peak at Rt- 5.20 min and chebulagic acid (2) peak at Rt - 12.15 min.

**Fig. 3:**
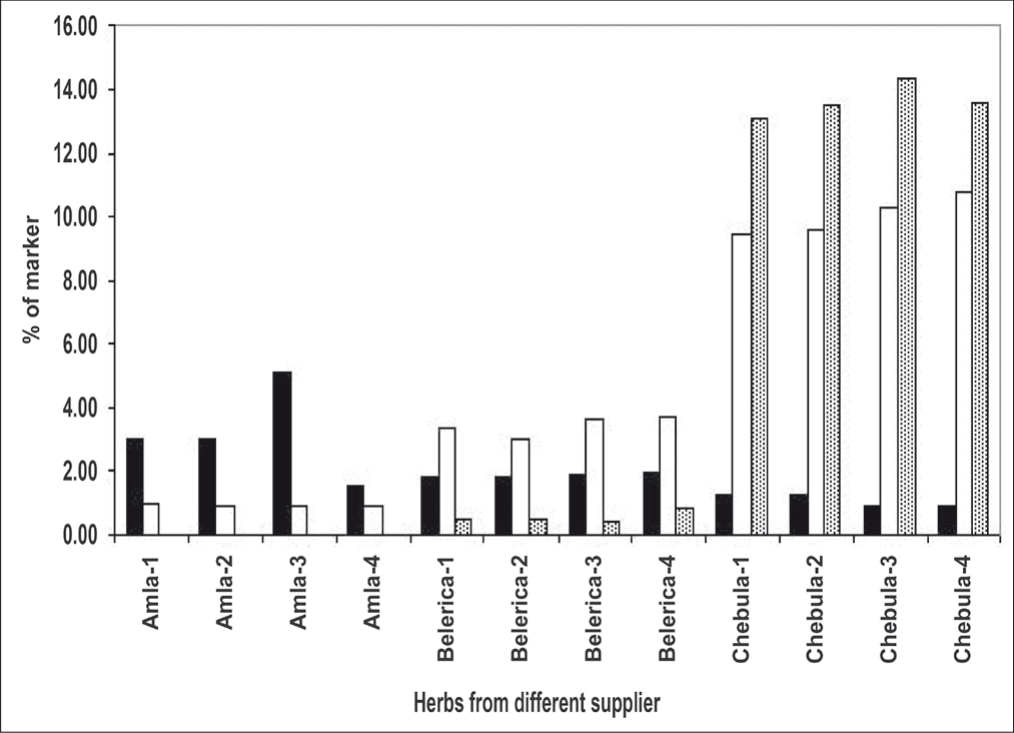
Histogram representing marker content (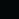) in individual herbs. Histogram shows gallic acid (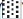), chebulinic acid (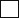) and chebulagic acid (□) content in individual herbs from four different suppliers.

*Amla* contains gallic acid in the range of 1.5-5% (w/w) and chebulagic acid is 0.9 to 1.2% (w/w), while chebulinic acid was not detected. In case of *belerica, chebula* and TC all three marker compounds are detected (fig. 2b, 2c and 2d) and amounts were quantified. In *belerica* samples under study, amount of gallic acid is around 1.8% to 2.4% (w/w), chebulagic acid varies from 2.9% to 3.75 (w/w) while chebulinic acid is in the range of 0.4% to 0.9%. For *chebula* samples, gallic acid content varied from 0.8% to 1.25%, chebulagic acid is between 9.4% to 10.8% (w/w) and chebulinic acid content varied from 13% to 14.3% (w/w) (fig. 3). Initially marker content in each of the myrobalans was estimated. The average values were computed and are given in Table 3.

**TABLE 3:** AVERAGE VALUES OF MARkER MOLECULES IN INDIVIDUAL HERBS

	gallic Acid*	chebulagic Acid*	chebulinic Acid*
*Amla*	3.01	0.91	--
*Belerica*	1.79	3.17	0.48
*Chebula*	1.24	9.52	13.29

The table gives percentage of each marker molecule in individual herb (*means average value n= 4). These values have been used for theoretical calculation of Ayurvedic and non Ayurvedic *Triphala* formulations as stated in Table 1.

**Fig. 4:**
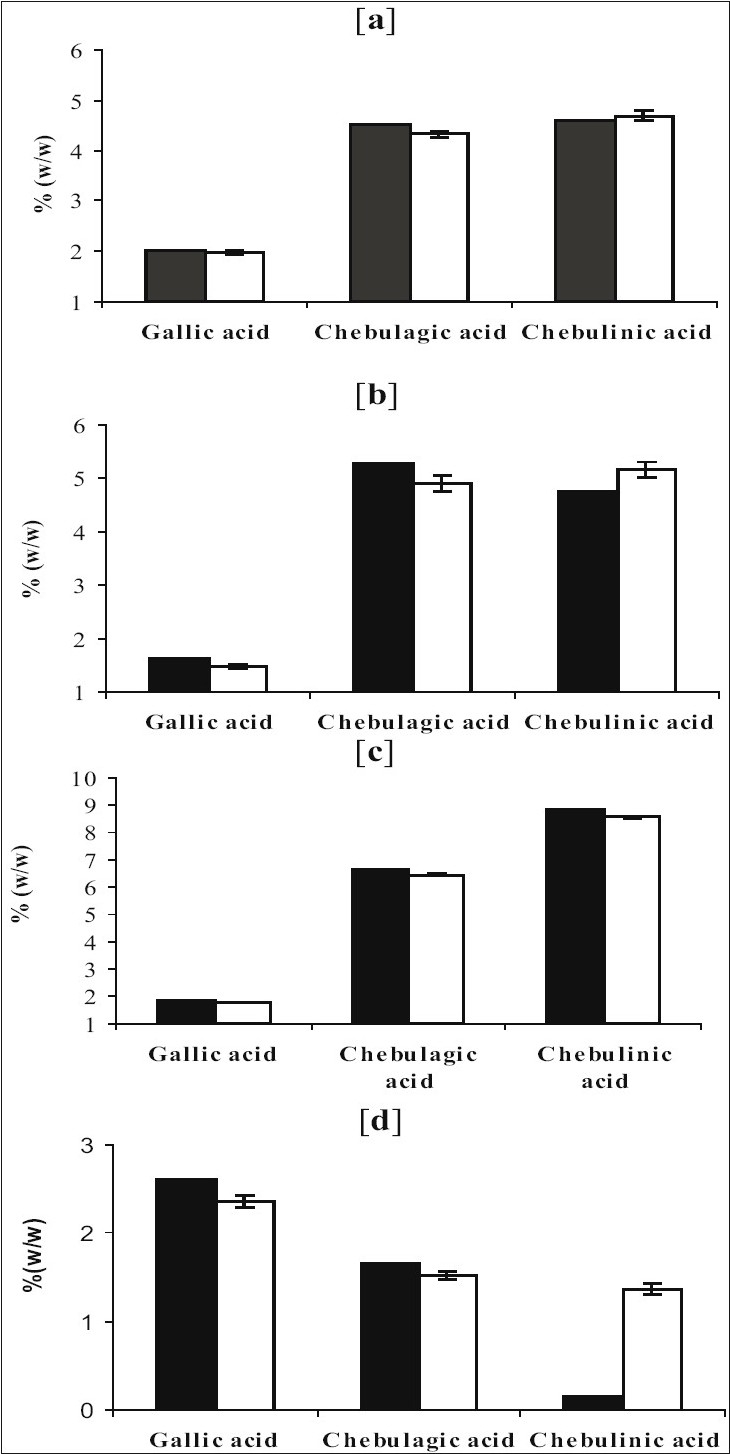
Histogram of theoretically calculated values of individual marker Vs Experimental observed value for Ayurvedic and non- Ayurvedic *Triphala* preparations. Histogram for *Triphala* formulation with *amla:belerica:chebula* ratios of a. (1:1:1), b. (0:2:1), c. (1:0:2) and d. (2:1:0). All histogram represents theoretical values of marker (■) and experimental values of marker (□)

Using (Table 3) theoretically, amount of gallic acid, chebulagic acid and chebulinic acid was calculated for *triphala* powder considering uniform mixing of *amla*, *belerica* and *chebula* in 1:1:1 ratio. The results of this theoretical calculation were compared with actual data obtained by HPLC analysis of TC blend prepared. These results were compared and given as a histogram (fig. 4a).

The histogram (fig. 4a) shows that there was only slight deviation from actual and theoretically calculated results. To confirm validity of our new approach, theoretical levels for each marker compound were calculated for all the non-traditional blends prepared as given in Table 1. For this the average values of individual herbs were taken from Table 2. Then using method developed the amount of marker compounds was estimated quantitatively. The theoretical values were comparable with the experimental values obtained (fig. 4b, 4c and 4d).

HPLC method validation data suggest that this HPLC method is accurate, precise, specific (match factor>90), with low limit of detection and quantification for all three markers, with high recovery values, The value of the regression coefficient (r^2^) of all three marker compounds were higher then 0.98, suggesting the linearity of the method. The method is rugged and robust.

There is no reported data on the uniformity of the composition of TC available in market. Possible variation can occur due to improper and inadequate mixing, variation in the particle size of each of the myrobalans, demixing during transportation and deviation from official textual (AFI)[1] composition. In case of a mixture of chemicals made of well-characterised synthetic molecules, uniformity of mixing can be tested by recovery experiments or by estimating proportions of each of the chemicals in the mix quantitatively. One of the critical challenges to achieve such testing is the natural variation in quantitative levels of the chemical compounds in herbs, which gets even more complicated when three herbs are blended together. An approach has been provided to test for the uniformity of mixing/composition during blending/manufacturing of *Triphala curna*, with the knowledge of each marker in the raw herb used for blending. This approach has potential to add variation data generation for GMP for product like TC with up to three herbs forming its composition.

## References

[1] (2003). Ayurvedic pharmacopoeia committee. The Ayurvedic Formulary of India, Part I.

[2] Juss SS (1997). Triphala – the wonder drug. Indian Med Gaz.

[3] Nadkarni AK (1976). Indian Materia Medica.

[4] Thakur CP, Thakur B, Singh S, Sinha PK, Sinha SK (1988). The Ayurvedic medicines, *haritaki, Amla* and *bahira* reduce cholesterol-induced atherosclerosis in rabbits. Int J Cardiol.

[5] Srikumar R, Parthasarathy NJ, Devi RS (2005). Immunomodulatory activity of Triphala on neutrophil functions. Biol Pharm Bull.

[6] Naik GH, Priyadarsini KI, Mohan H (2006). Free radical scavenging reactions and phytochemical analysis of *Triphala*, an Ayurvedic formulation. Curr Sci (India).

[7] Jagetia GC, Baliga MS, Malagi KJ, Sethukumar KM (2002). The evaluation of the radioprotective effect of *Triphala* (an Ayurvedic rejuvenating drug) in the mice exposed to gammaradiation. Phytomedicine.

[8] Jagetia GC, Rao SK, Baliga MS, Babu K (2004). The evaluation of nitric oxide scavenging activity of certain herbal formulations *in vitro*: A preliminary study. Phytother Res.

[9] Bahulikar AS, Kashalkar RV, Pundlik MD (2003). HPLC in standardization of herbal drugs: studies on *Triphala* powder. Asian J Chem.

[10] Choudhury RP, Kumar A, Garg AN (2008). Elemental characterization of *Triphala* powders and tablets by instrumental neutron activation analysis. J Herbal Pharmacotherapy.

[11] Mukherjee PK, Rai S, Bhattacharya S, Wahile A, Saha BP (2008). Marker analysis of polyherbal formulation, *Triphala*: A well known Indian traditional medicine. Indian J Traditional Knowledge.

[12] Singh DP, Govindrajan R, Rawat AKS (2008). High-performance Liquid Chromatography as a tool for the chemical standardization of *Triphala*: An Ayurvedic formulation. Phytochem Anal.

